# A qualitative exploration of knowledge, attitudes and practices of hospital pharmacists towards adverse drug reaction reporting system in Lahore, Pakistan

**DOI:** 10.1186/s40545-018-0143-0

**Published:** 2018-07-19

**Authors:** Rabia Hussain, Mohamed Azmi Hassali, Furqan Hashmi, Maryam Farooqui

**Affiliations:** 1 School of Pharmaceutical Sciences, Universiti Sains Malaysia, Penang, Malaysia; 2 University College of Pharmacy, University of the Punjab, Lahore, Pakistan; 3 Unaizah College of Pharmacy, Qassim University, Buraydah, Saudi Arabia

**Keywords:** Adverse drug reaction, ADR reporting system, Pharmacovigilance, DRAP, Barriers, Hospital pharmacist, Medication safety

## Abstract

**Background:**

Medication safety is a major public health concern and there are well established pharmacovigilance programmes in developed countries. However, there is scarcity of literature on the issue in low and middle income countries. In this context, the current study was aimed to evaluate the knowledge, attitudes and practices of hospital pharmacists towards medication safety and ADR reporting in Lahore, Pakistan.

**Methods:**

A qualitative approach was used to conduct this study. A semi-structured interview guide was developed, 10 hospital pharmacists were recruited and interviewed through convenience sampling technique. All interviews were audio-taped, transcribed verbatim, and were then analyzed for thematic contents analysis.

**Results:**

Thematic content analysis of the interviews resulted in 6 major themes, including (1) Familiarity with medication safety & adverse drug reaction concept (2) Current system of practice and reporting of adverse drug reaction in hospital setting, (3) Willingness to accept the practice change (4) Barriers to adverse drug reaction reporting, (5) Policy change needs and (6) The recognition of the role. Majority of the hospital pharmacists were familiar with the concept of medication safety and ADR reactions reporting however they were unaware of the existence of national ADR reporting system in Pakistan. Several barriers hindering ADR reporting were identified including lack of awareness and training, communication gap between the hospitals and regulatory authorities.

**Conclusion:**

The study revealed that that hospital pharmacists were good in understanding of medication safety and ADR reporting; however they don’t practice this in real sense. The readiness of the hospital pharmacist towards the practice change has indicated that they are all set to be actively involved in the provision of medication safety in hospital setting. Involvement of key stake holders from ministry of health, academia, pharmaceutical industry and healthcare professionals is warranted to promote safe and effective use of medicines.

## Background

Adverse drug reactions (ADR) represent a significant patient safety concern after being recognized as major cause of morbidity and mortality in hospital admissions [1]. World Health Organization (WHO) defines an ADR as, “a response which is noxious and unintended, and which occurs at doses normally used in humans for the prophylaxis, diagnosis, or therapy of disease, or for the modification of physiological function” [2].

As real-life use of drug is enormously different from the controlled clinical trial due to various reasons. It does not cover the associated effects of drugs for a longer duration of time, a its not a representative of some other populations such as children, elderly and people with different sets of morbidities in those trials [3]. To overcome these problems, the post marketing surveillance is considered as the best tool to recognize the possible effects of a drug [3]. Post marketing surveillance comes under pharmacovigilance (PV) which according to WHO is “the science and activities relating to the detection, assessment, understanding and prevention of adverse effects or any other possible drug-related problems” [4].

WHO established its first pharmacovigilance center in Uppsala, Sweden after the Thalidomide tragedy in 1961 [5]. Now it has collaborating centers all around the world. A pharmacovigilance (PV) center collects reports on possible ADR to detect the ADRs in the post marketing phase [6]. This spontaneous reporting is considered as the most important feature of the system whereby the reports are submitted to the national reporting agency through the healthcare professionals and pharmaceutical manufacturers. These reports are then communicated to WHO pharmacovigilance center [7]. Suspected ADRs reports from member countries of the WHO Programme for International Drug Monitoring are sent to the WHO international database ‘VigiBase’, which is managed by the WHO Uppsala Monitoring Centre UMC. The reports are reviewed and analysed and the evidence based recommendations are forwarded to the member countries [8].

In UK, according to Yellow Card scheme [9], every year almost 17,000 ADR reports are reported and hospital pharmacists in UK are officially responsible to report ADRs [10]. In US, several major national programs are working on pharmacovigilance including MedWatch, Sentinel Events Reporting Program and Medication Error Reporting Program, whereby the reporting involve both healthcare professionals and public [10–13]. In Canada, Canadian Adverse Drug Reaction Monitoring Program regulates the pharmacovigilance activities and pharmacist are part of this program since its inception [10, 14]. The Centre for Adverse Drug Reactions Monitoring (CARM) monitors adverse drug reactions in New Zealand, and pharmacists are also a part of this programme [15]. In Netherlands, a spontaneous ADR reporting scheme was launched in the 1963, and since then pharmacists have been involved in reporting. ADR reports are submitted by doctors and pharmacists to the Netherlands Pharmacovigilance Centre, and out of these reports about 40% reporting is done by the pharmacists [16]. Contrary to this, ADR reporting is scant in many low and middle come countries and it has impact on use of medicines, patient safety and on policy and practice [17].

### Adverse drug reaction monitoring in Pakistan

Pakistan is a lower middle-income country with a population of 207.8 million and ranked as the sixth most populous country in the world [18, 19]. In Pakistan, the healthcare system is comprised of three-tier structure, including primary, secondary and tertiary care [20]. Primary and secondary care centers cater the basic health needs of the population while tertiary care centers involve hospitals based modern facilities [20]. Most of the budget in Pakistan goes to tertiary health care centers [21] and the Ministry of National Health Services Regulation and Coordination (NHSRC) regulates the health system of the country [18].

There was no established pharmacovigilance system in the country until 2011, when a locally manufactured cardiac drug (Isotab 20 mg (Isosorbide mononitrate, batch number J093) caused death of more than 200 patients and hospitalization of 1000 patients in Lahore, Punjab [19, 22]. It turned out that the drug was adulterated and a serious error in quality assurance contributed to this incident [22]. As a result of this incident, Supreme court of Pakistan ordered the federal government to establish an independent drug regulatory authority [23]. To fulfill the order, in 2012, Drug Regulatory Authority of Pakistan (DRAP) was established under DRAP act 2012 [23]. Drug Regulatory Authority of Pakistan (DRAP) is one of the six divisions of Ministry of National Health Services, and Regulation (NHSRC) [23], which regulates the availability, quality, and safety of therapeutic goods including medical devices and medicines in the country [24]. After the establishment of DRAP things have started to get better, few hospitals in the country have pharmacovigilance in place, however overall the pharmacovigilance needs further improvement [25]. Drug Regulatory Authority of Pakistan (DRAP) in collaboration with international bodies including United States Pharmacopoeia and Promoting Quality Medicines (USP-PQM) has developed a framework to carry out post-marketing surveillance of drugs in Pakistan. Drug Regulatory Authority of Pakistan is also planning to obtain the membership of WHO Uppsala centre to access ‘Vigiflow’, Which is the global database system for pharmacovigilance reporting [26]. This access will help the medical professionals and drug regulators to stay updated with latest safety information about the drugs [27].

Punjab is the most populous province of the country comprising of half of the country’s population of 110 million [28]. Lahore is the provincial capital (of the Punjab province) and has a population of over 10 million people [29]. The Punjab Government is the first provincial government to establish provincial office of the pharmacovigilance Centre at the Directorate General Health Services, Punjab [30, 31]. The provincial drug control unit Punjab (PDCUP) is setting up policies and procedures regarding the aspects related to the drug control, it is also publishing medicine safety alerts on regular basis, and since its operation to date total 91 medicine safety alerts have been published [32]. Irrespective of the fact that the data is being updated on regular basis, still the communication gap lies between drug regulatory authorities and the healthcare professionals.

Pharmacists world over are playing an important role to promote the safe use of medicines, however in Pakistan studies on medicines use and pharmacovigilance are scarce. In this context, the present study is being planned to investigate the knowledge, attitudes and practices of hospital pharmacists on Adverse Drug Reaction reporting in Lahore. The study will use qualitative methods to explore attitude, practices and will help to fill the gaps in the literature. The information obtained from this article would be helpful to plan interventions to improve medicines safety.

## Methods

### Study design

We adopted a qualitative approach (social sciences research methods technique) to collect the data [33]. We opted this design due to certain reasons as the design is flexible and it allows an in-depth understanding that further help in better understanding of participants’ experiences and attitudes [34, 35]. Besides, the approach is helpful to generate a number of ideas that how individuals perceive the problem. In addition, qualitative research methods help to identify the gaps that cannot be otherwise identified by the survey based research methods [35, 36].

### Study setting

The study setting was Lahore. As it is a metropolitan city of the province of the Punjab (Pakistan) [29]. It has a population of over 10 million and is also a hub for culture, business and healthcare facilities [29, 37].

### Development of interview guide

Based on in depth review of literature [38–54] and current practices of hospital pharmacist in Pakistan, a semi structured interview guide was developed. The guide was designed in a way to explore the knowledge about medication safety and ADR reporting and its system. Perceptions and confidence about practice change and future interventions towards improvement in practices were also documented. Prior to data collection, the guide was tested for validity and reliability. The questionnaire was validated by two experienced academicians and researchers at the Universiti Sains Malaysia, Penang, Malaysia. Reliability of the study was assured by face to face interviews with the participants. The guide was piloted on 2 hospital pharmacists (who were excluded from the actual study). The guide was modified accordingly on the basis of pilot results. After obtaining the consent from the experts and interviewees, the interview guide was made available for the study (Appendix).

### Respondents and inclusion criteria

Hospital pharmacists working in tertiary care public hospitals as a full time, regular employees were selected for interviews. Contract based hospital pharmacists or pharmacists working in primary or secondary care public or private hospitals were excluded from the study. The study was given the approval by Humans Ethics Committee (HEC), University College of Pharmacy, University of the Punjab, Lahore, Pakistan with reference no. HEC/PUCP/1943.

### Sampling and data collection

Pharmacists in our study were purposively selected by using convenience sampling technique [55]. Prior to the participation in the interview, an explanatory statement detailing the objectives of the study was given to each hospital pharmacists and a written consent was obtained from them. Participant’s personal information was collected by the self-administered questionnaire attached with the consent form. The interview was conducted by the principal author of this study. The principal author had training in conducting qualitative interviews. The interviews were conducted at a time and place convenient for the participating pharmacist, mostly at the work place of the participants.

The interviews were conducted in English as pharmacy graduates can speak the language and this is the medium of education in the country. Each interview session lasted for 30–40 min. To seek required information where necessary, appropriate probing questions were asked from the respondents and additional field notes were taken. The interviews were audio recorded and transcribed verbatim by the researcher (RH). Data saturation was achieved after 8th interview, however 2 additional interviews were conducted to see if new themes were emerging. The researcher manually analyzed the transcripts line by line for relevant themes and content. The transcripts were independently coded by two researchers (RH and AH). Coding was compared, and consensus was obtained by the co-authors in the study.

## Results

### Demographics of the participants

A total of 10 hospital pharmacists aged between 25 and 38 years were interviewed. There were 8 female participants and 2 male participants (in Pakistan, female hospitals pharmacists are in majority, they prefer to work in hospital-based setting). Majority of the participants (*n* = 7) were from the age group of 31–40. Six participants had an experience of 1–5 years, while four pharmacists had an experience of 10 or more years of service. Eight of the participants had specialization in their field as Masters or PhD, while two participants were graduates. The demographic distribution of the participants is described as below (Table 1):

**Table 1:** Demographics of the respondents

Characteristics	Frequency
Gender	
Male	2
Female	8
Age (Years)	
20–30	3
31–40	7
> 40	0
Education	
Graduation	2
Specialization	8
Experience (years)	
1–5	6
6–10	2
> 10	2
ADR reporting	
Yes	2
No	8

### Thematic analysis

Thematic content analysis of the interview resulted in 6 major themes. These themes are listed as (1) Familiarity with medication safety & ADR concept (2) Current system of practice and reporting of ADR in the hospital setting (3) Willingness to accept the practice change, (4) Barriers to ADR reporting (5) Policy change needs and (6) Recognition of the role of pharmacists in the reporting.

### Theme 1: Familiarity with medication safety and ADR concept

During the interviews, the respondents were asked about their knowledge on medication safety and ADR concept.

*“Medication safety means that the medicine which you are giving to the patient, should be appropriate, right route of administration, right dose, and drug should be chosen in such a way that there will not be any harm to the patient.” (HP-4)*

*“To protect the patients from harm, from the adversities of the drug related products.” (HP-7)*

When asked about the concept of ADR, most participants came up with the standard definition by WHO [1]. This showed that the participants had good knowledge about the ADR.

*“Adverse drug reactions are the reactions that are abnormal, noxious and can cause life-threatening problems.” (HP-1)*

*“Effects come out which are not coming in a normal dose., allergic reaction due to some idiosyncrasy or something like that for the dose, that was not supposed to produce those effects, which might be due to individualized differences in metabolism…they just occur.” (HP-5)*

*“Any noxious, unintended drug reactions, which even occur at normal dose.” (HP-6)*

The hospital pharmacists were also asked about the type of ADRs they will prefer to report and there was mixed response related to this question. Some participants emphasized that only *major* and *severe* ADRs can be reported because they thought that minor ADRs can be handled and they do not require reporting. While some described that even a minor ADR should be reported.

*“Obviously major or moderate must be reported, minor can be tackled within the hospital.” (HP-2)*

*“Any suspected reactions should be reported, it can be a headache for one person, but for other person, it can be a severe thing.” (HP-3)*

### Theme 2: Current system of practice and reporting of ADR in hospital setting

The participants discussed about the current system of reporting of ADR in their work settings. The views by participants focused on the lack of interest of hospital administration in the hospital regarding practice of ADR reporting.

*“Mainly, it’s in my mind, because I never reported any ADR report on any form anywhere, its mainly in the conversation, technical discussions, have in meetings all the time instead of reporting. Because again it’s not a requirement by the hospital.” (HP 9)*

*“If patient came with an ADR, then our special team for emergency cases deals with them, we are not involved as such. After the stabilization of patient, we get a report from the ward, then we look if it’s a medication error or an adverse drug reaction, but no reporting as such on official basis.” (HP 10)*

### Theme 3: Willingness to accept the practice change

All hospital pharmacists were positive towards improvement in medication safety related practices. They related the practice change with their job satisfaction and quality of life of the patients.

*“I feel very positive and I think we should improve the practice and I am satisfied with my job as I am helping my patients.” (HP 1)*

*“It must be patient oriented, we need a positive change regarding strict monitoring of patient related parameters. I think system will benefit the patient, our main goal is to achieve wellness of patients.” (HP 2)*

*“Yes the quality of life of patients will be improved because on the basis of experience you can be more careful choosing the medicines, you have the information based on past experiences.” (HP 4)*

### Theme 4: Training needed to improve ADR reporting

Majority of the hospital pharmacists had no formal training on ADR reporting, although they had basic knowledge during their undergraduate degree program, however there was no implementation in hospital setting. For the better delivery of healthcare services, the hospital pharmacists pointed out that there is a need of necessary training.

*“We don’t have proper knowledge, training, proper system, don’t have access to published data. So, we need more training and awareness to understand what an adverse drug reaction can be…. there is a dire need to change the system.” (HP 8)*

*“They must train a lot of people on their agendas and get the patient safety work done, new protocols must be designed based on international guidelines. A system that can be very vigilant and very aggressive one, I must say.” (HP 9)*

### Theme 5: Barriers related to ADRs reporting

There were many barriers to under reporting of ADRs. Majority respondents stated that they did not report ADRs due to a number of issues including lack of knowledge and training, lack of time, lack of support from colleagues, lack of communication between healthcare professionals and the absence of a reporting mechanism.

*“It is difficult actually, I cannot give much time and cannot see the patients individually….and hindrance comes from the attitudes of staff nurses, as they really do not inform pharmacist about any ADR plus there is no ADR reporting system and nobody is serious about that…so, it’s difficult for me to report any ADR.” (HP-1)*

*“The key barrier is actually the burden which is on every appointee pharmacist, since there are only a few people appointed by government sector. So, you know by the administration we are actually not prioritizing clinical pharmacy as a priority. This is the problem….it makes reporting difficult because of the burden, lack of training and lack of prioritization.” (HP-9)*

The participants further identified lack of encouragement, overly burdened staff, legal liability as a barrier to ADR reporting.

*“Barriers are the lack of encouragement, unavailability of ADR reporting form, although individual efforts are there. Lack of time and training is there, legal matter can be involved in reporting an ADR.” (HP-4)*

A systematically functioning reporting system is absent, though the respondents had an idea that what and when to report but they had no idea where to report.

*“I don’t think so that there is some particular ADR reporting system. As we do not have any knowledge and also maybe there is a lack of interest by government that we are not aware about the existence of the ADR reporting system in Pakistan.” (HP-1)*

*“In files there is a body, yes…DRAP… but in working, it’s not functional as such. They have launched the ADR forms, but they have no mechanism to receive the reports and after receiving we don’t know what they are doing with those reports.” (HP-3)*

*“Being regulatory authority of Pakistan, they must have approached us, they must have trained us. We never received any email, training or even a circular.” (HP 9)*

It was interesting to note that the senior pharmacists and peers do not encourage reporting. This was reported as another barrier when pharmacists were interviewed.

*“Honestly, pharmacist will never report ADR, because whenever I discuss with any senior about any ADR, they all replied to me the same, that…don’t do this, this will blame your position, you will be blamed by hospital authority, and company leaders, so better to stay within your limits.” (HP 7)*

### Theme 6: Recognition of the role as custodian of medicine safety

Majority of the hospital pharmacists were of the view that the healthcare system can be improved, if the role of pharmacist is recognized by the organization, thus medicine safety will be improved.

Participants also expressed their willingness to accept this role as in leadership capacity.

*“So, in my opinion if role of pharmacist is implemented well then, no medicine related issue will be there because pharmacist can really take care of safety of medicines.” (HP-1)*

*“I feel, it’s now a need of healthcare team, that role of pharmacist should be considered as important. Because, once you are involved in healthcare team, you are at a good position and because you have a good knowledge of medicines right from the selection till the administration, so can advise healthcare team very well. So, I think now role is much more important and the responsibility level is also increased.” (HP-4)*

*“I think pharmacist has a key role in enhancing medicine safety. So, pharmacist have to be visible, be with the medicines.” (HP 7)*

## Discussion

The present study has revealed gaps in the ADR reporting in Pakistan. Though our respondents had a fair knowledge about medication safety and ADR reporting, however the majority were unaware about the existence of any such reporting mechanism in the country. This shows that the pharmacy curriculum is catering the needs of the undergraduate pharmacists to understand the issues. However, the necessary practical training to understand the ADR reporting was found deficient when it came to a practice. This lacking can be attributed by the traditional class room methodology, which is typically followed in Pakistani education system [56]. There is a need to train the pharmacy graduate in real setting by providing the opportunities like internships, trainings and seminars at undergraduate level. Moreover, WHO Uppsala Monitoring Centre (UMC) provides a number of web-based lectures, as well as it also offers distance learning course on signal detection and causality assessment on adverse drug reaction reporting for students as well as for healthcare professionals [17].

In our study we found that the hospitals have basic policies to report and monitor ADR reaction in the setting, but the practice varies from hospital to hospital. Looking at the Pakistani scenario, each provincial government is responsible for the regulation of healthcare facilities in their own province [57]. For example, hospitals in Punjab are regulated by health department, Government of Punjab and it is assumed that all of these hospitals are providing the uniform health care services to the population, however the situation in other provinces may be different [20]. In our study it was observed that the policy implementation related to ADR was not consistent, even within a single hospital, it was observed that different departments were implementing policies differently. As many of the hospitals even do not report a single case, others do report to the provincial health commission or even so to the national pharmacovigilance centre. It was also observed that many public hospitals are still unaware about this online portal and activities by DRAP. Although, The Drug Regulatory Authority of Pakistan has provided a web portal for reporting [24], still the under-reporting of ADR cases is a huge concern. The government officials and policy makers need to form and implement a uniform system regarding the reporting of adverse drug reactions nationwide.

The findings from our study also showed that the hospital pharmacists had a positive attitude towards practice change. This behavior pattern can be explained by employing Trans Theoratical Model (TTM) of change [58]. This model explains the behavior change of an individual and his readiness to accept a new healthier behavior. The model is comprised of six stages of behavior change which develops over a span of time [58]. As the present study focusses on the practices of pharmacist and their readiness to accept if any change comes to the way, therefore the model is believed to be a better explanation of the participants’ behavior. The hospital pharmacists working in Lahore had a mixed behavior towards the practice change. The study has revealed that that the pharmacist who were aged between 31 and 40 and had an experience of more than 10 years were at their pre-contemplation stage, while those who were aged between 21 and 30 with an experience of 5 years were in their contemplation stage. So, the newly graduated pharmacist should be trained properly and government bodies need to take interest in the continuous education and training of pharmacists to make them ready for the preparation phase of behavior change.

During the interviews, several barriers were identified which can impact on the reporting of an ADR. Among them workload is a key barrier to the ADR reporting. In Pakistani public hospitals, on every 1200 beds, a pharmacist is appointed, this differs to the world standard of one pharmacist for 50 beds [59]. The government is still unable to meet the minimum health delivery standards [59]. This can be related to 6000 vacant positions of hospital pharmacists in Punjab which are awaiting approval from government. Only 279 hospital pharmacists posts have been sanctioned by the government of the Punjab in last 10 years [60, 61]. This number is so few to cater the needs of a big population. Moreover, many of the public hospitals do not have a clearly defined job structure for the hospital pharmacists. The results from our study showed that most hospitals are not engaging pharmacists to provide clinical services, rather the appointed pharmacists are usually involved with the procurement and supply of drugs at ward level. These findings urge to revise the job structure of pharmacist including pharmacovigilance activities as the responsibility of the pharmacist.

It was noteworthy to state that pharmacist had a basic idea about pharmacovigilance, but majority of the interviewee pharmacist had no idea about the national pharmacovigilance centre by DRAP. Though, already there are few hospital pharmacists in public hospitals, however even they are not aware of the existence of national pharmacovigilance center. This raises questions regarding the communication between healthcare professionals, DRAP and its pharmacovigilance unit. In developed countries, authorities have created effective ways of communication which are both agile and engaging at the same time. Without dynamic and effective communications with key stakeholders of pharmacovigilance it is impossible to achieve the safe use of medicines which requires constant awareness and vigilance [62].

Another identified barrier was a lack of information technology and computertised record system in the public hospitals. The participants shared their concerns that due to the lack of computerized system in hospital, patient records cannot be updated, there is no tracking for the prescription orders and hence no ADR records are available. This strengthens the need for the establishment of an information technology based system in the country where the hospital computers could be linked. If there is a central database then reports from these hospitals can be sent to the national reporting center (DRAP) without any delay. Besides, mobile phones application could be developed and use of social media could also help with the time management issues related to under reporting [63].

During the interviews, hospital pharmacist emphasized on their role as medication safety expert, this is in line with the literature [64] . Another interesting finding came out from participants was the recognition of their roles as the focal person in case of ADR reporting [41, 65]. Majority of the pharmacists were of the view that the medicine safety will improve, if the role of pharmacist is recognized by the organization in which they work [66]. These finding are further strengthened by a Spanish study which pointed out the importance of pharmacist’s role in ADR reporting [67].

The respondents in our study also gave suggestions regarding the role of Drug Regulatory Authority of Pakistan to improve the ADR reporting in the country. This includes training and continuing education to health care professionals, providing time for reporting, acknowledging the effort in the form of certificates, incentives and publications. These suggestions were found to be similar with the other incentives reported in the literature [44, 68]. Though the government has already taken many major initiatives to improve ADR reporting including establishment of national pharmacovigilance centre, development of web portal and collaboration with USP-PQM, however the ADR reporting in real time is still not in place. The implementation of drug monitoring is needed both at individual and institutional level to make this programme a success [69]. A strong political will is also important to ensure the multidisciplinary collaboration involving ministry of health, academia, pharmaceutical industry and healthcare professionals regarding safe and effective use of medicines.

### Limitations of the study

The study was conducted in Lahore. Though it’s the second largest city in the country with a population of about 10 million, however results cannot be generalized to the whole country. There were only 10 hospital pharmacists who were interviewed in the study. Though the number is small however the study is qualitative in nature resulting in useful themes on the issue.

## Conclusion

The study showed that the hospital pharmacists were aware regarding medication safety and ADR reporting, however they don’t practice this in real sense. Pharmacists showed willingness to change practice provided barriers to ADR reporting could be removed. Identification of barriers by pharmacists are useful to design intervention towards better pharmacovigilance and adverse drug reporting mechanisms. Overall, these findings can serve as primer for policy makers and government to build future work in this area.

## Appendix

### Hospital Pharmacist’s interview guide

#### Objective: Exploring the knowledge, attitude & perceptions of hospital pharmacists towards medication safety & ADR reporting

**Part I**

**Focus: Knowledge and perceptions about Medication safety**

1. What comes to your mind when you hear the word “Medication Safety”?
2. Do you counsel patients on medication safety? If yes which particular aspect of it,under what conditions do you counsel them and why? (condition mean disease state)
3. How long the average counselling/ conversation takes when giving information about medication safety? Do you have enough time and how has this impacted on your workload/practice?

**Part II**

**Focus: Knowledge and attitudes about Adverse Drug Reaction (ADR) reporting**

1. What is your understanding of Adverse Drug Reaction?
2. What would you do if you were approached by a patient with a severe ADR (any recent incidence, what was your strategy to deal with the patient)?
3. What type of adverse drug reactions you consider should be reported?
4. In your current practice, how many ADR cases you have seen? (Did you report/ record them by yourself or heard it from some other colleagues)
5. Do you have any guidance on reporting or how to and when to report any ADR? And report to whom and Where?
6. Have you ever sent an adverse drug reaction report to your national reporting agency, when that happened and why did you send it?
7. Have you ever sent an adverse drug reaction report to the responsible pharmaceutical company, when that happened and why did you send it?’
8. Do you think that ADR reporting will influence the quality of life of the patients under your care?
9. In your opinion do you think that ADR reporting contributes to drug safety? If yes, (How)?
10. What are the factors that you think can impact and may encourage pharmacists to report ADRs (why a pharmacist should report an ADR)?
11. In your opinion, what are the possible factors that contribute as the barriers to ADR reporting?
12. Do you think your job in any way makes it easy/ difficult to report ADR.
13. What type of medicine information resources (journal, news, formulary{BNF}) you prefer while reporting ADR?
14. Do you receive and routinely review publications to become aware of medications with error potential?

**Part III**

**Focus: Knowledge about Adverse Drug Reaction reporting system**

1. Are you aware about the existence of the regulatory body that regulates ADR reporting in Pakistan? Reasons (if not aware)
2. Do you have any idea about the difference of medication safety and ADR reporting systems between Pakistan and other developed countries?
3. In your opinion, do you think that there is a need to change the system about medicine safety and ADR reporting? What benefit would it have?

**Part IV.**

**Focus: Future perspective**

1. What do you think that with the advancements in pharmacy practice, your role has changed and how it can contribute towards medication safety?
2. What could be the possible suggestions to improve ADR reporting (in hospital) in future? Any suggestions?
3. **Conclusion/suggestions**

Would you like to provide any additional comments about medication safety and ADR reporting system in Pakistan?

## Abbreviations

ADR: Adverse drug reaction
DRAP: Drug Regulatory Authority of Pakistan
HP: Hospital pharmacist
NHSRC: National Health Services Regulation and Coordination
PV: Pharmacovigilance
UMC: Uppsala Monitoring Centre
WHO: World Health Organization
